# Teaching skills for medical residents: are these important? A narrative review of the literature

**DOI:** 10.1590/1516-3180.2018.0147060818

**Published:** 2018-12-13

**Authors:** Saadallah Azor Fakhouri, Lorena Pinho Feijó, Kristopherson Lustosa Augusto, Maria do Patrocínio Tenório Nunes

**Affiliations:** I MD. Doctoral Student, Universidade Federal de Uberlândia (UFU), Uberlândia (MG), Brazil.; II MD. Professional Master’s Student, Centro Universitário Christus (UNICHRISTUS), Fortaleza (CE), Brazil.; III MD, PhD. Adjunct Professor, Department of Clinical Medicine, Faculdade de Medicina da Universidade Federal do Ceará (FAMED - UFC) and Universidade de Fortaleza (UNIFOR), Fortaleza (CE), Brazil.; IV MD, PhD. Associate Professor, Department of Internal Medicine, Faculdade de Medicina da Universidade de São Paulo (FMUSP), São Paulo (SP), Brazil.

**Keywords:** Education, medical, Internship and residency, Education

## Abstract

**BACKGROUND:**

There is extensive evidence, mainly from the United States and Canada, that points towards the need to train medical residents in teaching skills. Much of the “informal curriculum”, including professional values, is taught by residents when consultants are not around. Furthermore, data from the 1960s show the importance of acquiring these skills, not only for residents but also for all doctors. ­Teaching moments can be identified in simple daily situations, like discussing a clinical situation with patients and their families, planning patients’ care with the healthcare team or teaching peers and medical students. The aim here was to examine the significance of resident teaching courses and estimate the effectiveness of these courses and the state of the art in Brazil.

**METHODS:**

We conducted a review of the literature, using the MEDLINE, PubMed, SciELO and LILACS databases to extract relevant articles describing residents-as-teachers (RaT) programs and the importance of teaching skills for medical residents. This review formed part of the development of a doctoral project on medical education.

**RESULTS:**

Original articles, reviews and systematic reviews were used to produce this paper as part of a doctoral project.

**CONCLUSIONS:**

RaT programs are important in clinical practice and as role models for junior learners. ­Moreover, these educational programs improve residents’ self-assessed teaching behaviors and teaching confidence. On the other hand, RaT program curricula are limited by both the number of studies and their methodologies. In Brazil, there is no such experience, according to the data gathered here, except for one master’s thesis.

## INTRODUCTION

Learning medicine is not a lonely journey and is no longer a passive act. Doctors at both junior and senior levels participate in this complex process, thereby facilitating learning and consolidating and updating knowledge daily. Santos et al.^1^ described facilitators as doctors who are more experienced and who thus help in the professional development of undergraduates, medical residents and their peers. These authors referred to the work of Vygotsky,^2^ for whom “the learning process comes from outside sources and is conceived through individual interactions with the world”. Furthermore, they postulated that the role of facilitators (preceptors) is to enable some situations in which apprentices’ knowledge assimilation and production becomes transformed. The preceptor’s main role is therefore to facilitate the acquisition of theory and skills by stimulating his/her pupils to make their own discoveries.

Residency is characterized by in-service learning, i.e. training during practice within a scenario in which residents may become role models, in accordance with statements based on educational strategies for teaching and learning. Therefore, it is crucial to balance teaching, learning and healthcare assistance.

The most remarkable characteristic of medical residency is its in-service training, in which teaching is integrated with practice scenarios, so as to build a model for physicians’ ideological, ethical and professional identity. The professional competence that is expected at the end of a medical residency program needs to go beyond technical knowledge. It also encompasses skills and attitudes that show effective team capabilities, leadership, communication skills, empathy, self-control and metacognition.^3^ Sternszus et al.^4^ investigated the importance of resident role models in the education and career choices of medical students, in a cross-sectional survey-based study. Their study was the first to illustrate that resident role models are perceived by medical students to be as important as role models formed by attending physicians, for their education.

Medical residents are an essential part of the workforce in most Brazilian hospitals. In teaching centers, because of the humdrum nature of daily tasks, there is an additional responsibility towards helping students and peers, to help them improve their knowledge and technical skills. Several authors have noted that the word ‘doctor’ comes from the Latin verb *docere*,^5^ meaning to teach, with the aim of highlighting how teaching is important for all medical professionals, including those under training.

American researchers have estimated that residents spend almost a quarter of their residency programs teaching others, even though they are undertrained for this purpose.^6^ Much of the “informal curriculum”, including professional values, is taught by residents.^7^ This process focusing on peer-to-peer cooperative learning remains poorly studied.

Development of clinical acumen through good clinical teaching is a key component of medical education. Few residents will come to postgraduate training with well-developed teaching skills or a sense of their relevance to student education, or with knowledge of the principles of adult learning and its theory and practice.^5^

Pioneering studies conducted in the United States and Canada have highlighted the relevance of training medical residents to provide them with teaching skills. These studies demonstrated that 20-25% of the residents’ working hours were spent on teaching activities,^6^^,^^8^^,^^9^ and that medical students attributed 30-85% of all the clinical theory that they acquired during their undergraduate programs to teaching given by their residents.^10^^,^^11^ All of this exchange of information is provided through long working hours and exhaustive periods on call. Despite all the information presented above, doctors, medical students and residents receive little or no formal instruction on how to teach.^10^^,^^12^^,^^13^

Both the American College of Graduate Medical Education^14^ and the Liaison Committee on Medical Education^15^ have recommended that formal instruction on teaching skills should be provided for medical residents. These activities have been deemed to be so important that Louie et al.^5^ described the development of residents as teachers in terms not only of a necessary personal obligation but also of a national priority.

The aim of the present review was to assess the significance attributed to programs on teaching skills for residents that have been described in the literature. We aimed to compare these results with findings in Brazil, in order to provide up-to-date conclusions and recommendations regarding this topic.

## METHODS

We conducted a review of the literature, using MEDLINE, PubMed, SciELO and LILACS databases to extract relevant articles describing residents-as-teachers (RaT) programs and the importance of teaching skills for medical residents. This review formed part of the development of a doctoral project on medical education. We searched the literature between January 2015 and December 2017, covering the period between 1970 and 2017.

The MeSH terms used in the search were “internship and residency”, “education” and “education, medical”. The key words used in the search included “residents”, “residents as teachers”, “residents-as-teachers”, “residents AND teachers”, “residents AND education”, “education” and “medical education”. The key words used for the search in Portuguese in LILACS and SciELO included “medicos residentes”, “medicos residentes AND educacao”, “medicos residentes AND ensino”, “educacao medica” and “ensino em medicina”. The articles identified as containing information regarding teaching skills for medical residency were selected for intensive review and were analyzed by two authors (SAF and MPTN). Searches of primary publications referenced in other articles were also included. These were selected for intensive review and were analyzed by two authors (SAF and MPTN).

## RESULTS

The search in LILACS and SciELO using Portuguese key words did not yield any results on the specific topic of formal residents-as-teachers programs, while the search in MEDLINE/PubMed yielded 213 articles, of which 44 were found to contain information regarding teaching skills for medical residency. The references in Portuguese that were included in this paper were extracted from the personal files of one author (MPTN).

### 1) Selection of relevant articles:

Table 1 describes the numbers of texts extracted from each database. The relevant publications included original articles, systematic reviews, critical reviews, randomized controlled trials, guidelines from medical education experts and material from pioneering authors in this field. Table 2 shows the most relevant studies included in this review and summarizes their main characteristics.

**Table 1.** Databases and numbers of articles extracted

Database	Number of articles
PubMed	68
MEDLINE	95
Other sources*	4

*Personal files of one author.

**Table 2.** Relevant studies on residents-as-teachers (RaT) programs that were included in this review

Authors	Year	Study type*	Participants (n)	Specialty	Main objectives	Conclusions
Jewett et al.^20^	1982	RCT	55 residents	Pediatrics	To compare residents who received clinical teaching instruction with those who did not.	The intervention group was significantly more confident as teachers and received more positive feedback on their teaching.
Sheets et al.^19^	1991	RL	NA	Surgery	To describe evidence that supported the importance of training surgery residents in teaching skills.	Residency program directors and faculty members within surgery needed to acknowledge that teaching was an important component of residents’ daily agenda.
Bordley et al.^22^	2000	NR	NA	Internal medicine	To describe the importance of teaching skills for residents and discuss the costs involved in these activities at different institutions.	The authors strongly supported investment in training residents as teachers.
Morrison et al.^13^	2000	NR	NA	NA	To describe the number of RaT programs in the US and demonstrate evidence for their implementation and evaluation.	Research was needed to identify the most appropriate design for RaT programs and how they affected educational outcomes.
Furney et al.^30^	2001	RCT	57 second- and third-year residents	Internal medicine	To compare residents who received a one-hour intervention based on the One-Minute Preceptor, with a control group.	Intervention group residents reported statistically significant changes in all behaviors after the One-Minute Preceptor.
Morrison et al.^37^	2002	QS	100 medicine students, residents and faculty members	Internal medicine Pediatrics Family medicine	To describe the learning needs of residents for becoming more effective teachers, using 11 focus groups and 4 semi-structured interviews.	Residents filled important roles as practical clinical teachers and role models for junior learners.
Wamsley et al.^26^	2004	RL	14 articles on RaT programs	Multiple	To examine the evaluation methods for resident teaching courses and estimate the effectiveness of those teaching courses.	Resident teaching courses improved resident self-assessed teaching behaviors and teaching confidence. Further studies were needed to elucidate the best format, length, timing and content of these courses and to determine whether they influenced learner performance.
Morrison et al.^23^	2005	QS	21 third-year residents	Internal medicine Family medicine Pediatrics	To compare residents who received a 13-hour training in teaching skills in the previous year, with those who did not, through semi-structured interviews.	Intervention group residents expressed more enthusiasm for teaching, learner-centered learning and self-knowledge about teaching. Control group residents seemed easily frustrated by time constraints and often expressed cynicism and guilt toward learners.
Dewey et al.^33^	2008	SR	13 articles on RaT programs	Multiple	To identify all randomized control trials (RCTs) on residents’ teaching skills programs in psychiatry.	Only one trial incorporating psychiatry residents was found to exist.
Busari et al.^21^	2009	QS	18 residents	Pediatrics Obstetrics and gynecology	To extract recommendations from interviews, regarding how a training program for residents could be created.	Enthusiasm and enjoying teaching were good attributes of successful teachers. Reasons for poor teaching were lack of time and absence of support from attending staff.
Post et al.^27^	2009	SR	24 articles on RaT programs	Multiple	To provide an updated systematic review of the literature on RaT program curricula and determine the most evidence-based curriculum and evaluation strategy.	Research on RaT program curricula was limited by both the number of studies and their methodology. The results demonstrated that these curricula can significantly improve residents’ teaching skills.
Karani et al.^18^	2014	QS	37 third-year medical students	NA	To describe what students learned from residents and teaching strategies used by excellent resident teachers.	In this study, role modeling was the most frequently classified teaching model. Residents’ teaching was critically important for undergraduate students.
Owolabi et al.^38^	2014	QS	20 residents	Internal medicine	To evaluate the clinical teaching skills of internal medicine residents from the perspective of medical students in a tertiary-level teaching institution in Africa.	Residents’ clinical teaching skills were suboptimal, particularly regarding their ability to promote understanding and retention.
Dannaway et al.^28^	2016	RL	12 articles on RaT programs	Multiple	To assess the current evidence regarding the efficacy of teaching skills programs for junior medical officers.	The review of the literature demonstrated many positive effects from teaching skills programs, thus supporting their use. Substantial threats of bias were present in most studies.
Ramani et al.^17^	2016	FG	NA	Multiple	To guide medical educators involved in the implementation of RaT programs.	The authors highlighted the importance of congruence between formal and hidden curricula and encouraged evidence-based approaches within education.
Al Achkar et al.^16^	2017	QQS	221 residency program directors	Multiple	To compare the number of RAT programs with data from a previous study in 2000 and ask for feedback about the importance of these activities.	Over 80% of the residency programs surveyed had implemented RaT programs. Program directors had realized that there was a clear need for formative training experiences for residents.
Chokshi et al.^32^	2017	IR	29 second-year residents	Pediatrics	The authors developed and evaluated an intensive one-day RaT program curriculum using a flipped classroom approach.	Residents demonstrated statistically significant improvements in performance between pre- and postworkshop evaluations through objective structural teaching evaluations and attitudinal and self-efficacy questionnaires.

*NR = narrative review; QQS = qualitative and quantitative survey; FG = framework guide; QS = qualitative study; RL = review of the literature; RCT = randomized controlled trial; SR = systematic review; IR = innovation report; NA = not applicable.

### 2) Residents-as-teachers programs from abroad:

Specific agendas for training residents in teaching skills were first developed in the 1960s. Since then, the numbers of residents-as-teachers (RaT) programs around the world has increased and their methodologies have improved. RaT programs are nowadays part of most residency programs in the United States.

Morrison et al.^13^ reported that the prevalence of programs for developing residents’ teaching skills in the United States was 55%. In 2017, the same authors published new data in which the prevalence was established as 80.54%, i.e. a 26.34% increase (95% confidence interval, CI: 20.39% to 32.29%) over the last 15 years.^16^ Ramani et al.^17^ described the potential benefits of RaT programs in the Association for Medical Education in Europe (AMEE) 2016 guide 106. Karani et al.^18^ reported that being a role model was the tool that residents who were teaching most frequently identified. Acquiring teaching skills was seen to involve complex conscious and unconscious activities, through observation and reflection on behaviors.

### 3) Impact of residents-as-teachers programs on residents and students:

RaT programs have been particularly successful for several reasons. Medical students like to work with residents and, additionally, appreciate their close supervision. Thus, residents are seen as a positive influence and example of professionalism.^10^^,^^19^^,^^20^^,^^21^ Moreover, residents spend large amounts of time together with medical students. They are close to them in practical activities and have similar ages and professional development processes. Most residents feel more satisfaction with their work while experiencing teaching duties.^22^^,^^23^ Moreover, these programs are especially attractive because they improve self-confidence in teaching.

Teaching by residents is different from and probably complementary to that of institutions’ attending staff and faculty members. Residents tend to teach

1. different things (bedside skills and patient management rather than factual knowledge);
2. in a different way (as near-peer teachers); and
3. at different times (teaching while on-call).^24^

In a qualitative analysis on a teaching initiation module that had been presented, it was concluded that it was possible to develop pedagogical skills at this stage, in a process that would be coherent with changes to health and education policies.^25^ According to the same authors, residents needed to learn:

1. to take on leadership and provide a role model;
2. to give guidance to learners;
3. to give feedback,
4. to teach bedside skills;
5. to teach about procedures;
6. to teach about inpatients;
7. to teach about charting; and
8. to give lectures.

Most of the studies on RaT programs have shown that residents achieved significant improvement in teaching skills after some specific training. The first review of the literature on this topic was published in 2004 and analyzed 13 studies with different experimental designs.^26^ A review conducted in 2009 assessed 24 studies on residents who were enrolled in several programs and found that in 21 of these studies there was significant enhancement of teaching performance after specific training.^27^ A recent assessment on 39 studies, published in 2016, demonstrated that interventions based on providing teaching skills up to level 3 on Kirkpatrick’s outcome scale were effective (Table 3).^28^^,^^29^

**Table 3.** Kirkpatrick’s model^29^ for evaluating educational outcomes*

Kirkpatrick level	Evaluation outcome	Explanation
Level 1	Reaction	Participants’ views of the learning experience and its organization, presentation, content, teaching methods and quality of instruction
Level 2A	Learning - change in attitudes	Changes in attitudes or perceptions among participant groups towards teaching and learning
Level 2B	Learning - modification of knowledge or skills	For knowledge, this relates to the acquisition of concepts, procedures and principles For skills, this relates to the acquisition of thinking and problem-solving, psychomotor and social skills
Level 3	Behavior - change in behavior	Documents the transfer of learning to the workplace or willingness of learners to apply new knowledge and skills
Level 4A	Results - change in the system or organizational practice	Refers to wider changes in the organization attributable to the educational program
Level 4B	Results - change among the participants: students and peers	Refers to improvement in medical student or peer learning or performance as a direct result of the educational intervention

*Adapted from Kirkpatrick (1994).^29^

The evidence supporting RaT programs appears not to correlate the time spent on training with efficiency in teaching. Even a one-hour intervention showed benefits in a study involving internal medicine residents.^30^ A four-week elective course created for senior and family medicine residents paved the way for a paper giving advice on how to create a RaT program.^31^ In another study published in 2017, 29 participants were enrolled in a successful one-day RaT program that used a “flipped classroom” approach.^32^

In another systematic review, an efficacy analysis was conducted on residents from a wide spectrum of specialties who participated in single programs.^33^ On the whole, the interventions and outcomes measured were heterogeneous and the quality of the methodologies varied. The authors felt that these programs brought the opportunity to advance educational research in this field.^33^

Residents who teach acquire the material that they teach more effectively than they would if they did it through self-study or through attending lectures.^34^ Their teaching duties have been linked to greater job satisfaction.^23^

In 2016, a meta-analysis revealed that most studies on residents-as-teachers programs had significant methodological flaws. Nevertheless, it was found that the main impacts of these interventions included improvement of attitudes and positive perceptions toward clinical teaching (Kirkpatrick level 2a); support for modification of knowledge or skills (Kirkpatrick level 2b); development of teaching skills (Kirkpatrick level 3) and some improvement of students’ learning after the intervention (Kirkpatrick level 4b). Some studies revealed positive organizational change (Kirkpatrick level 4a).^28^

Residents with better teaching skills seem to have greater knowledge of taught material and better clinical skills. In a retrospective study on senior residents in general surgery (covering the period from 2009 to 2013), technical ability was assessed through their performance in the Fundamentals of Laparoscopic Surgery examination. Teaching ability was assessed through evaluations among medical students on a Likert scale. There was evidence that residents who were better teachers had greater knowledge of taught material and a higher degree of laparoscopic skills.^35^

Snell^24^ provided information on the effects of RaT programs on residents. This author showed that those who were teaching had greater enthusiasm for teaching and greater job satisfaction.

Residents with effective teaching skills may also have a positive effect on patient care. Involvement of residents in teaching activities has been shown to have a positive effect on their communication skills, and good patient communication skills have been associated with better clinical outcomes.

### 4) Content of current RaT programs:

RaT programs have been delivered using many methods, including lectures, small-group discussion, practice with peers, videotape reflections and role-playing. The total number of hours dedicated to RaT program instruction varies widely. In the USA, RaT programs are more prevalent within pediatrics, family medicine, internal medicine, psychiatry, obstetrics/gynecology and surgery.

A thematic analysis identified five main reasons for implementing RaT programs: (1) teaching is part of residents’ role; (2) learners desire formal RaT training; (3) regulatory bodies require RaT training; (4) RaT programs improve residents’ education; and (5) RaT programs prepare residents for their current and future roles. There are also five reasons for not implementing RaT training: (1) lack of time and energy; (2) lack of expertise and resources; (3) newness of the program; (4) limited access to students; and (5) RaT instruction is not desired.^16^

### 5) Brazilian context:

A recent review of the literature only identified one study on RaT programs in Brazil.^36^ The authors concluded that there was no description of formal development of teaching skills within medical residency curricula, according to the databases that were searched. Regarding the teaching-learning process (for residents in family medicine), the authors stated that the pedagogical project and teaching plan for RaT programs constituted a social complex that would need a reflective, critical and collaborative approach.

## DISCUSSION

This narrative review shows that residents with better teaching skills might have greater knowledge of taught material and better clinical skills, according to some studies, which in turn enhances patient care. Students and senior doctors agree that residents have a critical role in the teaching process.^37^ Residents themselves can also recognize their protagonist role in spreading knowledge to students, peers, patients and families.

Around the world, universities set the goals for preparing residents to teach. These objectives have been seen as an effective way of improving the entire educational experience of residents and preparing them for the future.

It is not just in developed countries that the importance of training residents in teaching skills is acknowledged. Some developing nations are also publishing their own experiences in this field. One study in Nigeria has highlighted the importance of bringing medical residents into the teaching scenario in countries with limited resources for hiring teachers.^38^

RaT programs are delivered using many methods and a great variety of lengths of training. However, the resources available, and especially the expertise to lead the instruction, are not distributed equally among residency programs.

Data on training residents as teachers in Brazil is scarce despite the creation of new medical schools and the resulting increase in the number of students, which has brought difficult challenges to the educational system. There is an urgent need for better preparation of educators for teaching. Since 2006, Hospital das Clínicas of the Federal University of Pernambuco (Universidade Federal de Pernambuco, UFPE) has offered a 64-hour training program to medical residents within family medicine, with the aim of transforming this reality.^36^

In the literature, it is stated that residents who teach have greater enthusiasm for teaching, with greater job satisfaction. Residents are also role models for medical students. It is possible that pedagogical development also has a positive effect on patient care, though addition of effects regarding physician communication skills, with better clinical outcomes

In an ongoing study, Brazilian residents at a public university are being trained in the One-Minute Preceptor^39^ methodology, in comparison with controls. Our training education program combines theoretical and practical resources with a unique one-hour long dynamic. The partial results are very encouraging, with good validation scores for peer feedback based on the Stanford Faculty Development Program 26 (SFDP-26) instrument.^40^ Our study also revealed that significant positive changes to the residents’ teaching skills were achieved.

One innovative facet of this study was that it included scarce data on RaT programs in Brazil. We aimed to explore the reasons for implementing RaT instruction, given the fact that most Brazilian programs for residency training currently do not include RaT programs. We face the challenge of teaching pedagogical strategies for medical residents in this country. The positive effects may be of great significance for students, medical residents and patients.

The present study was susceptible to selection bias and reporting bias. We also only explored instruction of RaT program mode that had been introduced. Hence, little can be concluded regarding the effectiveness of any other form of instruction or the relevance of any other targeted skills.

## CONCLUSIONS

It is necessary to add pedagogical training to the training for residents and others working in the Brazilian National Health System, regarding ethical, technical and scientific knowledge. Some successful initiatives for developing skills and attitudes within healthcare education have emerged. However, these are still insufficient.

The necessary expansion of medical residency programs and the already inflated number of medical schools in Brazil both require qualified teachers. Implementing pedagogical training during residency training could improve clinical skills and patients’ care.

An accurate diagnosis regarding the state of pedagogical attributions among medical residents in Brazil is urgently necessary. There is also a need to understand what tools are at their disposal to perform the act of teaching with quality and efficacy, cooperatively.

Considering the positive effects that have been demonstrated, it can be argued that all residency programs should require residents to undergo instruction relating to teaching skills.

Along with teacher development programs, training for medical residents as teachers has the capacity to boost the quality of healthcare education in Brazil.
